# PAR2-mediated upregulation of BDNF contributes to central sensitization in bone cancer pain

**DOI:** 10.1186/1744-8069-10-28

**Published:** 2014-05-05

**Authors:** Yanju Bao, Wei Hou, Rui Liu, Yebo Gao, Xiangying Kong, Liping Yang, Zhan Shi, Weidong Li, Honggang Zheng, Shulong Jiang, Conghuang Li, Yinggang Qin, Baojin Hua

**Affiliations:** 1 Department of Oncology, Guang’anmen Hospital, China Academy of Chinese Medical Sciences, Beixiange 5, Xicheng District, 100053 Beijing, China; 2 Institute of Chinese Materia Medica, China Academy of Chinese Medical Sciences, Nanxiaojie, Dongzhimen District, 100700 Beijing, China; 3 Department of Nephrology, Guang’anmen Hospital, China Academy of Chinese Medical Sciences, Beixiange 5, Xicheng District, 100053 Beijing, China; 4 Department of Oncology, Jining First People’s Hospital, Healthy Road 6, 272000 Shandong, China

**Keywords:** Proteinase-activated receptor 2, Brain-derived neurotrophic factor, Nuclear factor-κB, Glutamatergic transmission, Bone cancer pain

## Abstract

**Background:**

Bone cancer pain is currently a major clinical challenge for the management of cancer patients, and the cellular and molecular mechanisms underlying the spinal sensitization remain unclear. While several studies demonstrated the critical role of proteinase-activated receptor (PAR2) in the pathogenesis of several types of inflammatory or neuropathic pain, the involvement of spinal PAR2 and the pertinent signaling in the central sensitization is not determined yet in the rodent model of bone cancer pain.

**Findings:**

Implantation of tumor cells into the tibias induced significant thermal hyperalgesia and mechanical allodynia, and enhanced glutamatergic strength in the ipsilateral dorsal horn. Significantly increased brain-derived neurotrophic factor (BDNF) expression was detected in the dorsal horn, and blockade of spinal BDNF signaling attenuated the enhancement of glutamatergic strength, thermal hyperalgesia and mechanical allodynia in the rats with bone cancer pain. Significantly increased spinal PAR2 expression was also observed, and inhibition of PAR2 signaling ameliorated BDNF upsurge, enhanced glutamatergic strength, and thermal hyperalgesia and mechanical allodynia. Inhibition of NF-κB pathway, the downstream of PAR2 signaling, also significantly decreased the spinal BDNF expression, glutamatergic strength of dorsal horn neurons, and thermal hyperalgesia and mechanical allodynia.

**Conclusion:**

The present study demonstrated that activation of PAR2 triggered NF-κB signaling and significantly upregulated the BDNF function, which critically contributed to the enhancement of glutamatergic transmission in spinal dorsal horn and thermal and mechanical hypersensitivity in the rats with bone cancer. This indicated that PAR2 - NF-κB signaling might become a novel target for the treatment of pain in patients with bone cancer.

## Introduction

Bone cancer pain is currently a major clinical challenge for the management of cancer patients, because the specific cellular and molecular mechanisms underlying bone cancer pain largely remain obscure [1,2]. While lines of studies have demonstrated the remarkable sensitization of peripheral nociceptors in the bone, resulting from the tumor-induced acidosis and cytokine synthesis, emerging evidences indicated that the activation of astrocytes [3-5] and altered glutamatergic synaptogenesis [6,7] existed in the spinal dorsal horn of the rodent model of bone cancer pain. Overwhelming evidences strongly suggest that brain-derived neurotrophic factor (BDNF) serves as one of the key regulators of synaptic plasticity in brain regions including cortex, hippocampus as well as spinal cord [8], and it is critically involved in the induction and maintenance of central sensitization in varieties types of pain [9]. It was previously reported that the synthesis and release of BDNF was remarkably upregulated, which contributing to the central sensitization, in the spinal dorsal horn of the rodent with inflammatory or neuropathic pain [9,10]. Currently, the role of BDNF in the induction and maintenance of enhanced spinal glutamatergic transmission has not been elucidated in the rodent model of bone cancer pain.

Proteinase-activated receptors (PARs) are a family of G-protein coupled receptors that are activated by proteases, which liberates a tethered ligand, by cleaving the N-terminus of the receptors, and initiates several intracellular signal pathways [11]. Four subtypes of PARs exist. While PAR1, PAR3, and PAR4 are preferentially activated by thrombin, PAR2 is preferentially activated by trypsin and trypsin-like proteinases [12]. All four PARs are expressed throughout the peripheral and central nerve system. In the spinal dorsal horn, PAR2 receptor is located in the microglia, astrocytes, neurons, and the terminals of afferent fibers originating from the dorsal root ganglions [12]. Previous studies demonstrated the critical involvement of PAR2 in the pathogenesis of several types of inflammatory or neuropathic pain [12]. Activation of PAR2 signaling participated in the induction of sensitization of peripheral nociceptors in the rodent model of bone cancer pain [13]. However, it remains uncertain whether activation of spinal PAR2 signaling is critically involved in the central sensitization in the rodent model of bone cancer pain.

Besides triggering the mitogen-activated protein kinases signaling, including ERK1/2, p38 and JNK activity, the activation of G-protein couple receptor PAR2 also involves PLC-mediated hydrolysis of phosphatidylinositol 4,5-bisphosphate (PIP_2_) and induction of the Ca^2+^/inositol 1,4,5-trisphosphate (IP_3_)/PKC signaling [11], which may in turn induce the phosphorylation of IKKα and IKKβ and result in the activation and nuclear translocation of NF-κB [11]. Accumulating evidence demonstrated that peripheral inflammation or nerve injury induced remarkable NF-κB activity in the spinal dorsal horn, which contributing to the thermal hyperalgesia and mechanical allodynia in the rodent models of chronic pain [14,15]. It was previously reported that upregulated NF-κB activity was required for the developmental and plasticity-associated synaptogenesis in the central neurons [16]. Meanwhile, upregulation of NF-κB activity also facilitated the expression of BDNF in the central neurons [17]. Here, the present study aims to elaborate the involvement of PAR2 - NF-κB signaling in the induction of enhanced spinal glutamatergic transmission and painful behaviors in the rats with bone cancer.

## Results

### BDNF contributes to the enhanced glutamatergic strength in the rats with bone cancer pain

**Figure 1:**
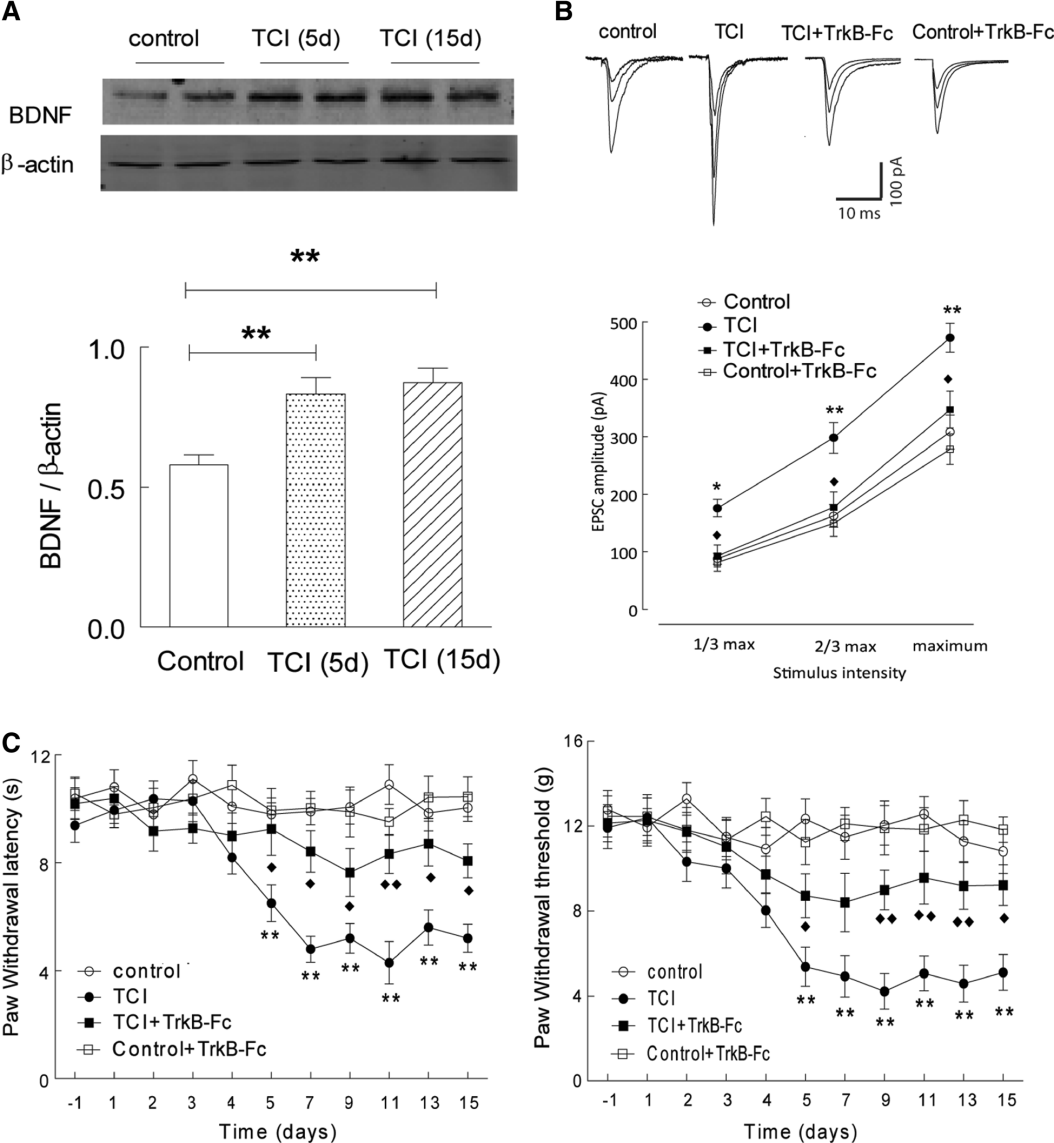
**Inhibition of BDNF signaling attenuated pain behavior in the rats with bone cancer. (A)** Implantation of tumor cells (TCI) into the tibias significantly increased the protein expression of BDNF in the spinal dorsal horn of the rats (day 5: t = 3.7, P < 0.01; day 15: t = 4.7, P < 0.01; n = 7 rats in each group); **(B)** Intrathecal injection of TrkB-Fc (1.5 μg, from day 3 through day 15) attenuated the increase of input (stimuli intensity) – output (EPSC amplitude) response in the dorsal horn neurons in the modeled rats (F_(1,20)_ = 13.36, P < 0.01, n = 9–12 neurons per group); **(C)** Intrathecal injection of TrkB-Fc significantly recovered the ipsilateral paw withdrawal threshold response to mechanical (F_(1, 18)_ = 11.24, P < 0.01) and radiant thermal stimuli (F_(1, 18)_ = 10.85, P < 0.01) in the rats with bone cancer (n = 8–10 rats per group). Control vs. TCI: *, P < 0.05; **, P < 0.01; TCI vs. TCI + TrkB-Fc: ♦, P < 0.05; ♦♦, P < 0.01.

Implantation of tumor cells into the tibias significantly decreased the ipsilateral paw withdrawal threshold responsive to the mechanical stimuli (Figure 1C), which was remarkably detectable at day 5, and maintained through at least day 15 (the endpoint of the study) after the procedure. Meanwhile, no significant change of the ipsilateral paw withdrawal threshold was observed in the rats receiving boiled cells. Similarly, the ipsilateral paw withdrawal latencies responsive to the radiant thermal stimuli were also significantly shortened in the rats implanted with tumor cells, which started at day 5 through day 15 (Figure 1C). These results indicated the bone cancer-induced mechanical allodynia and thermal hyperalgesia in the modeled rats.

To determine the adaptation of glutamatergic transmission in the spinal dorsal horn of the rats with bone cancer pain, the whole cell-recording was performed in the ipsilateral laminae II neurons in spinal slices (L4-L5), and the glutamatergic EPSC was recorded in the presence of strychnine (2 μM) and bicuculline (10 μM). The EPSC was completely abolished by the perfusion of CNQX (10 μM) and APV (10 μM), confirm its glutamatergic components. Compared to that in the control rats, the input (intensity of stimuli) –output (amplitude of evoked EPSC) response was significantly left shifted (Figure 1B), indicating a significantly enhanced glutamatergic strength in the dorsal horn neurons in the rats with bone cancer pain.

BDNF, synthesized from central neurons and astrocytes, is critically involved in the glutamatergic synaptogenesis and synaptic plasticity in the brain [8]. Here, we investigated the expression of BDNF in the dorsal horn, and its functional significance in the bone cancer-induced pain. As shown in Figure 1A, the expression of BDNF was significantly increased in the dorsal horn of the rats with bone cancer pain. The further electrophysiological recording studies revealed that intrathecal delivery of TrkB-Fc chimera (1.5 μg, day 3 to day 15), which quenching the endogenous BDNF, significantly ameliorated the increase of glutamatergic strength in the dorsal horn neurons of the rats with cancer-induced pain (Figure 1B). Behavioral studies demonstrated that inhibition of BDNF signaling by TrkB-Fc chimera also significantly attenuated the mechanical allodynia and thermal hyperalgesia in these modeled rats (Figure 1C). Note that blockade of the BDNF signaling failed to remarkably change basal glutamatergic transmission and pain behavior in the control rats (Figure 1B and C). These results suggested the critical involvement of BDNF signaling in the enhanced glutamatergic transmission in the dorsal horn neurons of the rats with bone cancer pain.

### PAR2 mediated BDNF upregulation and glutamatergic sensitization in the rats with bone cancer pain

**Figure 2:**
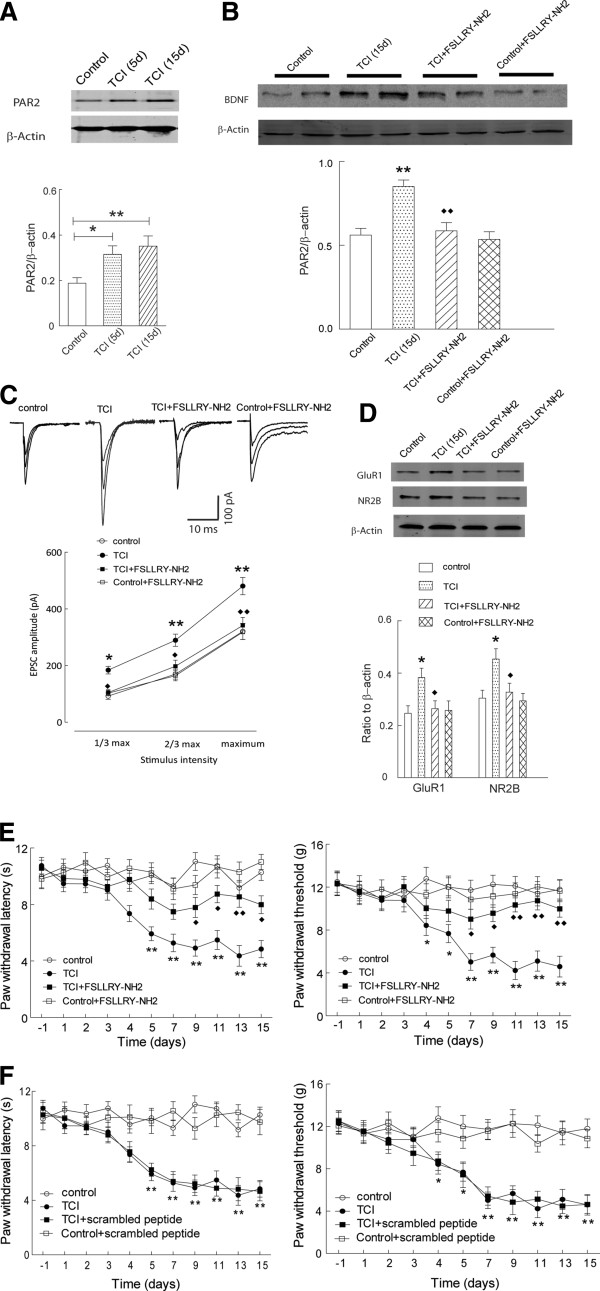
**Inhibition of PAR2 signaling attenuated pain behavior in the rats with bone cancer. (A)** Implantation of tumor cells in the tibias significantly increased the expression of PAR2 in the spinal dorsal horn of the rats (day 5: t = 2.84, P < 0.05; day 15: t = 3.18, P < 0.01; n = 7 rats in each group); **(B)** Blockade of PAR2 signaling by FSLLRY-NH2 (10 μg, from day 3 through day 15) significantly attenuated the upregulation of spinal BDNF in the rats with bone cancer pain (t = 4.21, P < 0.01, n = 7 per group), while no change was observed in the control rats (t = 0.42, P > 0.05, n = 6–7 rats per group); **(C)** Intrathecal injection of FSLLRY-NH2 significantly attenuated the increase of input (stimuli intensity) – output (EPSC amplitude) response in the dorsal horn neurons in the modeled rats (F_(1, 21)_ = 10.75, P < 0.01, n = 9–12 neurons per group); **(D)** Intrathecal injection of FSLLRY-NH2 significantly attenuated the increase of GluR1 (t = 2.57, P < 0.05) and NR2B (t = 2.40, P < 0.05) in the dorsal horn in the modeled rats (n = 6–7 rats per group); **(E)** Intrathecal injection of FSLLRY-NH2 significantly recovered the ipsilateral paw withdrawal threshold response to mechanical (F_(1, 18)_ = 10.04, P < 0.01) and radiant thermal stimuli (F_(1, 18)_ = 12.49, P < 0.01) in the rats with bone cancer (n = 8–10 rats per group); **(F)** Intrathecal injection of scrambled peptide failed to change the pain behavior in control and modeled rats (P > 0.05, n = 8–10 rats per group). Control vs. TCI: *, P < 0.05; **, P < 0.01; TCI vs. TCI + FSLLRY-NH2: ♦, P < 0.05; ♦♦, P < 0.01.

Considering the important role of PAR2 in neuroinflammation and the pathogenesis of several kinds of pain [12], we next determine the involvement of spinal PAR2 signaling in the induction of the enhancement of spinal glutamatergic transmission and pain behavior in the rats implanted with tumor cells. Firstly, as shown in Figure 2A, the expression of PAR2 was significantly increased in the dorsal horn of the rats with bone cancer. This indicated the potential role of PAR2 in the pathogenesis of bone cancer-induced pain.

Then, FSLLRY-NH2 (10 μg, from day3 to day 15), the antagonist of PAR2, was daily delivered via the intrathecal catheter to determine its effect on BDNF function, glutamatergic strength and pain behavior in the rats with bone cancer. As shown in Figure 2B, blockade of PAR2 signaling by FSLLRY-NH2 significantly reversed the upregulation of spinal BDNF in the rats implanted with tumor cells, while it did not obviously change the expression of spinal BDNF in the control rats. This suggested a PAR2-mediated spinal BDNF upregulation in the setting of bone cancer pain.

Further recording results demonstrated that, as shown in Figure 2C, intrathecal delivery of FSLLRY-NH2 significantly attenuated the enhanced glutamatergic input–output response in the dorsal horn neurons of the rats with bone cancer, while it did not remarkably change the basal glutamatergic strength in the control rats (Figure 2C). It was also found that intrathecal delivery of FSLLRY-NH2 attenuated the increased expression of glutamate receptor subunits GluR1 and NR2B in the dorsal horn of the rats with bone cancer (Figure 2D). As well, behavioral studies also demonstrated that inhibition of PAR2 signaling by FSLLRY-NH2 significantly attenuated the mechanical allodynia and thermal hyperalgesia in these modeled rats (Figure 2E). Notably, intrathecal injection of scramble peptides in the same dose failed to affect the mechanical and thermal pain behaviors in either control or modeled rats (Figure 2F). These results suggested that upregulation of PAR2 signaling, via modulating the expression of BDNF, critically participated in the induction of enhanced spinal glutamatergic transmission and pain behavior in the rats with bone cancer.

### NF-κB mediated BDNF upregulation and glutamatergic sensitization in the rats with bone cancer-induced pain

**Figure 3:**
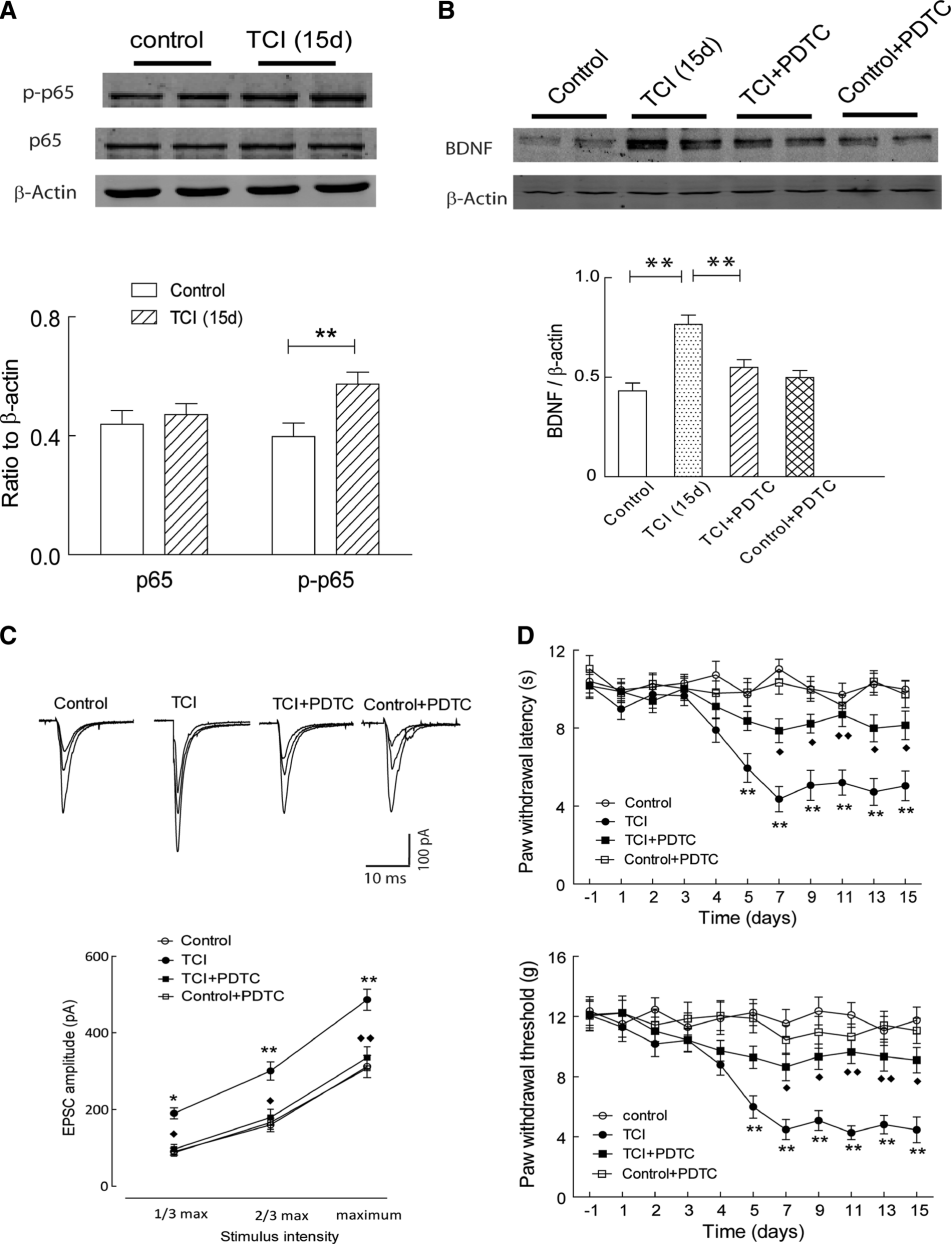
**Inhibition of NF-κB signaling attenuated pain behavior in the rats with bone cancer. (A)** Implantation of tumor cells in the tibias significantly increased the phosphorylation of NF-κB subunit p65 (t = 2.93, P = 0.01), but not the total p65 (t = 0.54, P > 0.05), in the spinal dorsal horn of the rats (n = 7 rats in each group); **(B)** Blockade of NF-κB signaling by PDTC (1 μg, from day 3 through day 15) significantly attenuated the upregulation of spinal BDNF in the rats with bone cancer-induced pain (t = 3.49, P < 0.01), while no change was observed in the control rats (t = 0.15, P > 0.05; n = 8–9 rats per group); **(C)** Intrathecal injection of PDTC significantly attenuated the increase of input (stimuli intensity) – output (EPSC amplitude) response in the dorsal horn neurons in the modeled rats (F_(1, 19)_ = 9.76, P < 0.01; n = 9–12 neurons per group); **(D)** Intrathecal injection of PDTC significantly recovered the ipsilateral paw withdrawal threshold response to mechanical (F_(1, 17)_ = 11.63, P < 0.01) and radiant thermal stimuli (F_(1, 17)_ = 9.81, P < 0.01) in the rats with bone cancer (n = 9–10 rats per group). Control vs. TCI: *, P < 0.05; **, P < 0.01; TCI vs. TCI + PDTC: ♦, P < 0.05; ♦♦, P < 0.01.

Activation of PAR2 signaling may induce the degradation of NF-κB inhibitor protein IkB, and lead to the activation and nuclear translocation of transcription factor NF-κB [18], which is critically involved in the synaptic plasticity [16] and processing of nociceptive information [19]. Here, as shown in Figure 3A, although the total expression level of NF-κB subunit p65 did not have significant change, the phosphorylated p65 was significantly increased in the ipsilateral dorsal horn of the rats implanted with tumor cells. Then, pyrrolidine dithiocarbamate (PDTC, 1 μg/10 μl from day 3 to day 15), an inhibitor of NF-κB, was daily delivered via intrathecal catheter to suppress the PAR2-NF-κB signaling in the rats with bone cancer pain. As shown in Figure 3B, blockade of NF-κB signaling by PDTC significantly reversed the upregulation of spinal BDNF in the rats with bone cancer-induced pain, while it did not obvious change the expression of spinal BDNF in the control rats. Further electrophysiological studies revealed that, as shown in Figure 3C, intrathecal delivery of PDTC significantly attenuated the enhanced glutamatergic strength in the dorsal horn of the rats implanted with tumor cells, while it did not remarkably change the basal glutamatergic strength in the control rats. Meanwhile, behavioral studies also demonstrated that inhibition of NF-κB signaling by PDTC significantly attenuated the mechanical allodynia and thermal hyperalgesia in these modeled rats (Figure 3D). These results suggested that activation of NF-κB signaling contributed to the PAR2-mediated BDNF upregulation, enhanced spinal glutamatergic transmission and pain behavior in the rats with bone cancer.

## Discussion

While inflammatory mediators and cytokines from peripheral nociceptors and surrounding immune and epithelial cells may remarkably sensitize the primary afferent fibers in the conditions of nociceptive and neuropathic pain [20], sensitization of nocisponsive neurons in spinal dorsal horn and supraspinal brain regions also significantly contributes to the persistent characteristics [21] and the negative affective components of sustained pain [22]. Peripheral inflammation may drive α-amino-3-hydroxy-5-methyl-4-isoxazolepropionic acid receptor subunit GluR1 membrane trafficking and induce hyperreactivity in the dorsal horn neurons [23]. Peripheral nerve injury also significantly enhanced the excitatory glutamatergic transmission in the dorsal horn neurons, which contributing to the mechanical allodynia in the neuropathic pain [24]. In the rodent model of bone cancer pain, the primary sensory afferents innervated in the bone marrow, mineralized bone, and the overlying periosteum was sensitized by the inflammatory microenvironment and injury of sensory and sympathetic fibers induced by proliferating tumor cells [2]. Meanwhile, emerging evidences recently indicated that sensitization of dorsal horn nocisponsive neurons existed in the rodents with bone cancer pain. Increased expression and phosphorylation of NR2B, an N-methyl-D-aspartate receptor (NMDA) subunit, was reported in the dorsal horn of the bone cancer pain models [25-27]. In the present study, a remarkably increased input (stimulation intensity) – output (EPSC amplitude) response was observed in the dorsal horn neurons, which substantiating the enhanced spinal glutamatergic strength and central sensitization in the rodent with bone cancer pain.

Overwhelming evidences demonstrated that BDNF extensively participated in the neuronal development and the induction and maintenance of synaptic plasticity in several brain regions [28]. As well, a growing body of evidence also suggested that BDNF was critically involved in spinal plasticity and central sensitization in varieties types of pain. Expression of BDNF was increased in the spinal dorsal horn of the rodent models of inflammatory or neuropathic pain [10]. Enhanced BDNF-mediated signaling induces the phosphorylation of NMDA receptor subunits and sprouting of spinal nocisponsive fibers [29], and enhances the communication between microglia and neurons in the dorsal horn [30], thus contributing to the induction and maintenance of the sensitization of spinal neurons in the circumstance of chronic pain. Here, in the present study, a significantly increased BDNF in dorsal horn was revealed, and blocking BDNF signaling largely attenuated the enhanced glutamatergic strength and painful behavior in the rodent with bone cancer pain. This suggested a critical involvement of BDNF in the spinal sensitization and hypersensitivity to painful stimuli in the bone cancer pain.

Upon activation, PAR2 receptor may trigger several intracellular signal cascades involving MAPK signaling, PLC-mediated phospholipid signaling, and β-arrestin signaling [11]. Among these, activation of PLC-mediated phospholipid signaling may induce the phosphorylation of IKKα and IKKβ and lead to activation and nuclear translocation of NF-κB [11,18]. Activation of NF-κB - mediated signaling increased the histone acetylation and facilitated the expression of BDNF in the central neurons [31]. Actually, it was recently implied that activation of PAR2 signaling was required for several inflammatory mediators-induced BDNF release from the microglia [32,33]. Moreover, the present study demonstrated that inhibition the activity of either PAR2 or NF-κB largely attenuated the upregulation of BDNF in the dorsal horn, indicating that the PAR2 - NF-κB signaling critically participate in the spinal BDNF upregulation in the bone cancer pain.

PAR2 is expressed in the primary sensory neurons and nocisponsive neurons in the spinal dorsal horn. Increasing evidences suggested that activation of PAR2 signaling was extensively involved in the sensitization of peripheral nociceptors and dorsal horn neurons in inflammatory and neuropathic pain. Activation of PAR2 and downstream enzymes PLC, PKCϵ, and PKA resulted in the sensitization of peripheral nociceptors by decreasing the threshold of transient receptor potential (TRP) ion channels in the circumstances of peripheral inflammation [34,35] or chemotherapeutic agent-induced neuropathic pain [36]. Chronic compression of dorsal root ganglion (DRG) can activate PAR2 signaling, leading to increased nociceptor hyperexcitability and thermal hyperalgesia in the rodents [37,38]. It was recently reported that activation of PAR2 signaling induced the sensitization of DRG neurons, which contributing to the thermal and mechanical hyperalgesia in the rodent model of bone cancer pain [12,13]. The present study further revealed that activation of PAR2 signaling remarkably contributed to the enhanced glutamatergic strength in the dorsal horn neurons, which implying its role in the induction of central sensitization in bone cancer pain. The present study also suggested that activation of NF-κB signaling may underlie PAR2-mediated BDNF upregulation, central sensitization and behavioral hypersensitivity in the bone cancer pain.

## Conclusion

The present study demonstrated that activation of PAR2 triggered NF-κB signaling and significantly upregulated the BDNF function, which substantially underlaid the enhancement of glutamatergic transmission in spinal dorsal horn and thermal and mechanical hypersensitivity in the rats with bone cancer pain. This indicated that PAR2 - NF-κB signaling might become a novel target for the treatment of pain in patients with bone cancer.

## Materials and methods

### Animal model of bone cancer pain and drug delivery

Adult female Wistar rats (weighing 200–250 g) were purchased from the Institutional Center of Experiment Animals, and were housed in the standard lab conditions (22 ± 1°C and 12:12 h light cycle) with unrestricted free access to food and water. All animal experiments were approved by the Institutional Animal Care and Use Committee in China Academy of Chinese Medical Sciences, and were in strict accordance with the recommendations in the Guide for the Care and Use of Laboratory Animals of the National Institutes of Health. All surgery was performed under appropriate anesthesia, and all efforts were made to minimize suffering in the animals.

The rat model of bone cancer pain was established by implantation of Walker 256 rat mammary gland carcinoma cells into the right tibias, as previously reported [3]. Briefly, suspensions of tumor cells in PBS was prepared as previously described [39]. Tumor cell implantation was achieved by injecting the tumor cells (1 × 10^5^ cells/μl, 5 μL) into the intramedullary space of the right tibia to induce bone cancer in rats under the anesthesia with pentobarbital (45 mg/kg). Boiled cells were injected with a similar procedure in the control group. The investigators who performed behavioral tests, immunoblotting, and electrophysiological recording were blinded to this procedure.

Intrathecal cannulation was performed as described previously [40]. Intrathecal catheters (PE-10 polyethylene tubing) were implanted 1 week before any procedures. The catheters were advanced 8 cm caudally through an incision in the cisternal membrane and secured to the musculature at the incision site. Only animals with no evidence of neurological deficits after catheter insertion were studied. TrkB-Fc chimera (1.5 μg, R&D systems cat#: 688-TK-100) [41], FSLLRY-NH2 (10 μg, Tocris cat#: 4751) [42], pyrrolidine dithiocarbamate (PDTC, 1 μg, Sigma cat#: P8765) [43], or vehicle was administered in a volume of 10 μl followed by a 10-μl flush with normal saline. The drugs were given daily from day 3 to day 15 after carcinoma cell inoculation.

### Behavioral tests

Mechanical allodynia and thermal hyperalgesia were assessed in rats to evaluate painful behavior induced by tumor cell implantation. Mechanical sensitivity was assessed by using a series of von Frey filaments with logarithmic incremental stiffness (Stoelting Co., Wood Dale, IL), and 50% probability paw withdrawal thresholds were calculated with the up-down method as previously described [44]. In brief, the animals were placed individually beneath an inverted ventilated cage with a metal-mesh floor, and allowed to habituation for 30 minutes before testing. The filaments were applied to the plantar surface of the hindpaws for approximately 6 seconds in an ascending or descending order after a negative or positive withdrawal response, respectively. Six consecutive responses after the first change in response were used to calculate the paw withdrawal threshold (in grams). The maximum pressure was set as 16 g.

Thermal nociceptive thresholds were measured using radiant heat [45]. Briefly, rats were placed into individual plastic cubicles mounted on a glass surface maintained at 30°C and allowed an hour habituation period. A thermal stimulus (a radiant heat source) was then delivered to the plantar surface of the hindpaws. The time for the rat to remove the paw from the thermal stimulus was electronically recorded as the paw withdrawal latency (PWL). The stimulus shut off automatically when the hind paw moved or after 16 seconds to prevent tissue damage. The intensity of the heat stimulus was maintained constant throughout all experiments.

### Protein extraction and immunoblotting

After behavioral testing, the rats were deeply anesthetized with pentobarbital sodium (50 mg/kg), and the ipsilateral L4-L5 segments were quickly removed and homogenized in the lysis buffer (25 mM Tris–HCl, pH 7.6, 150 mM NaCl, 0.1% SDS, 1 mM PMSF, 1 mM NaF, 1 mM NaVO_3_, 1 μg/ml leupeptin, 1 μg/ml pepstatin, and 1 μg/ml aprotinin). The lysates were centrifuged at 14,000 rpm for 10 min at 4°C, and the protein concentrations in the supernatant were measured by using the Bio-Rad protein assay kit. The samples (containing 20 μg proteins) were separated on a 15% (for BDNF) or 7.5% (for other proteins) SDS-polyacrylamide gel, and blotted to a nitrocellulose membrane. The blots were incubated overnight at 4°C with a rabbit polyclonal anti-BDNF primary antibody (1:250; Santa Cruz Biotechnology cat#: sc-546), monoclonal anti-PAR2 antibody (1:1000; Millipore cat#: MABF243), polyclonal anti-NF-κB p65 (1:1000, Abcam cat#: ab7970), anti-phosphorylated NF-κB p65 (1:1000, Abcam cat#: ab106129), or monoclonal anti-β-actin antibody (1:2000; Santa Cruz Biotechnology, cat#: sc-81178). The membranes were washed extensively with Tris-buffered saline and incubated with horseradish peroxidase-conjugated anti-mouse and anti-rabbit IgG antibody (1:10,000; Jackson ImmunoResearch Laboratories Inc., West Grove, PA). The immunoreactivity was detected using enhanced chemiluminescence (ECL Advance Kit; Amersham Biosciences). The intensity of the bands was captured digitally and analyzed quantitatively with Image J software. The immunoreactivities of target proteins were normalized to that of β-actin.

### Spinal cord slice preparation and electrophysiological recording

The rats were deeply anesthetized with inhalation of halothane, and the lumbar segment of the spinal cord was removed through laminectomy. The spinal tissue was immediately placed in ice-cold artificial cerebrospinal fluid containing (in mM): sucrose 230, KCl 3.5, MgCl_2_ 1.5, CaCl_2_ 2.0, NaH_2_PO_4_ 1.2, glucose 12, and NaHCO_3_ 25. Transverse spinal cord slices (400 μm) were cut with a vibratome (Technical Products International, St. Louis, MO), and incubated in Krebs’ solution (containing: 117 mM NaCl, 3.6 mM KCl, 1.2 mM MgCl_2_, 2.5 mM CaCl_2_, 1.2 mM NaH_2_PO_4_, 11 mM glucose, and 25 mM NaHCO_3_, bubbled with 95% O_2_ and 5% CO_2_) at 35°C for at least 1 h before the recording was performed.

Whole-cell recording on the spinal cord slices (L4-L5) was performed as described previously [40]. The neurons in lamina II of the ipsilateral dorsal horn were visualized using an upright microscope with infrared illumination (BX50WI; Olympus, Tokyo). Whole-cell voltage-clamp recordings were performed using an Axopatch 200B amplifier (Molecular Devices) with 3–5 MΩ glass electrodes containing the following internal solution (in mM): K-gluconate, 126; NaCl, 5; MgCl_2_ 1.2; EGTA, 0.5; Mg-ATP, 2; Na_3_GTP, 0.1; HEPES, 10; guanosine 5-*O*-(2-thiodiphosphate) 1; lidocaine *N*-ethyl bromide (QX314), 10; pH 7.3; 290 – 300 mOsmol. A seal resistance of ≥ 2 GΩ and an access resistance of 15 – 20 MΩ were considered acceptable. The series resistance was optimally compensated by ≥70% and constantly monitored throughout the experiments. The membrane potential was held at -60 mV throughout the experiment. Excitatory postsynaptic currents (EPSCs) in ipsilateral lamina II neurons were evoked by electrical stimulation (0.25 ms, 0.05 – 0.3 mA) of the dorsal root in the presence of strychnine (2 μM) and bicuculline (10 μM). The evoked EPSCs were filtered at 2 kHz, digitized at 10 kHz, and acquired and analyzed using pCLAMP 9.2 software (Molecular Devices).

### Statistical analysis

All data were presented as means ± SEM. For analysis of immunoblotting data, differences between groups were compared by Student’s *t*-test or ANOVA followed by Fisher’s PLSD post hoc analysis. The electrophysiological and behavioral data were compared with two-way ANOVA with repeated measurements. The criterion for statistical significance was *P* < 0.05. Statistical tests were performed with SPSS 13.0 (SPSS, USA).

## Competing interests

The authors declare that they have no competing interests.

## Authors’ contributions

YB, WH, CL, YQ, LY and RL performed the animal modeling and behavioral studies, and contributed to the manuscript. YB, YG, and ZS performed the electrophysiological recording, and YB, WL, HZ and XK performed the immunoblotting study. LY and SJ participated in the data analysis and coordination. BH and WH designed the study, supervised the experiments, analyzed the data and finalized the manuscript. All authors have read and approved the manuscript.
